# A Method for Capture and Detection of Crop Airborne Disease Spores Based on Microfluidic Chips and Micro Raman Spectroscopy

**DOI:** 10.3390/foods11213462

**Published:** 2022-11-01

**Authors:** Xiaodong Zhang, Fei Bian, Yafei Wang, Lian Hu, Ning Yang, Hanping Mao

**Affiliations:** 1 School of Agricultural Engineering, Jiangsu University, Zhenjiang 212013, China; 2 Key Laboratory of Modern Agricultural Equipment and Technology, Ministry of Education, Jiangsu University, Zhenjiang 212013, China; 3 Key Laboratory of Key Technology on Agricultural Machine and Equipment, Ministry of Education, South China Agricultural University, Guangzhou 510640, China; 4 School of Electrical and Information Engineering, Jiangsu University, Zhenjiang 212013, China

**Keywords:** micro Raman, microfluidic chip, fungal spores, crop disease, numerical simulation

## Abstract

Airborne crop diseases cause great losses to agricultural production and can affect people’s physical health. Timely monitoring of the situation of airborne disease spores and effective prevention and control measures are particularly important. In this study, a two-stage separation and enrichment microfluidic chip with arcuate pretreatment channel was designed for the separation and enrichment of crop disease spores, which was combined with micro Raman for Raman fingerprinting of disease conidia and quasi identification. The chip was mainly composed of arc preprocessing and two separated enriched structures, and the designed chip was numerically simulated using COMSOL multiphysics5.5, with the best enrichment effect at W2/W1 = 1.6 and W4/W3 = 1.1. The spectra were preprocessed with standard normal variables (SNVs) to improve the signal-to-noise ratio, which was baseline corrected using an iterative polynomial fitting method to further improve spectral features. Raman spectra were dimensionally reduced using principal component analysis (PCA) and stability competitive adaptive weighting (SCARS), support vector machine (SVM) and back-propagation artificial neural network (BPANN) were employed to identify fungal spore species, and the best discrimination effect was achieved using the SCARS-SVM model with 94.31% discrimination accuracy. Thus, the microfluidic-chip- and micro-Raman-based methods for spore capture and identification of crop diseases have the potential to be precise, convenient, and low-cost methods for fungal spore detection.

## 1. Introduction

Crop diseases cause huge losses to agricultural production and directly affect the economic development and national food security of many countries in the world [1]. Among them, fungal diseases can cause huge losses to the growth and yield of crops. In addition, fruits will still be damaged by fungi after being picked [2]. Fungal diseases mostly exist in the form of spores before infecting crops and fruits. In addition to causing damage to crops and fruits, fungal spores can also enter the lungs through the human respiratory tract and spread to other organs of the human body, causing various fungal diseases [3,4].Therefore, it is necessary to capture and identify megaspores quickly and accurately.

The identification and counting of spores under the traditional microscope mainly depend on naked eye observation. Due to the large number of spores captured, this method is labor-intensive, time-consuming, and inefficient. The accuracy of observation depends on the professional experience of operators, sometimes leading to large errors. Image processing methods are used to automatically detect and count spores, including image segmentation using K-means clustering algorithm, recognition based on shape factor and area, and spore contour segmentation based on concavity and contour segment merging. The automatic detection of disease spores has good effectiveness and accuracy, but spore recognition based on image processing technology cannot contain too many features [5,6]. Using deep neural network FSNet to detect fungal spores can automatically identify and count fungal spores in microscopic images, but image and deep learning methods are still not accurate enough to identify spores with similar shape and size [7]. PCR is the gold standard method for microbial detection. It is often used for microbial identification with high accuracy. However, this method requires professionals to crack the spores under strict experimental conditions. Only through tedious processing can fungal spores be detected [8,9]. The existing microscopic image method can realize rapid detection and recognition through morphology, but it cannot accurately identify spores with similar shape and size. The PCR method has high accuracy, but the detection conditions are harsh, destructive sampling is required, and the timeliness is poor, and the cost is high. Therefore, it is urgent to develop a rapid and accurate spore detection and identification technology. Raman spectroscopy is a light scattering technology with low cost and high speed. It can reflect various vibration frequencies and related vibration levels of biological components and can be used to identify the molecular composition and structure of biological samples [10,11]. Micro Raman spectroscopy has been applied to the identification and analysis of bacteria, which can be classified and identified [12,13]. However, the content of spores in the air is low, and there are a lot of impurities, so it is difficult to detect them directly using Raman spectroscopy. Therefore, a separation and enrichment method is needed to achieve spore detection and improve detection accuracy.

In recent years, microfluidic technology has provided powerful tools for detection applications due to its portability, miniaturization, automation, multi-channel sample detection, and cost saving. Compared with traditional methods, the greatest advantage of microfluidic control is to create a controllable microenvironment, which can accurately drive and control the microfluidic flow in the microchannel, thus improving the detection sensitivity [14,15]. Among them, the impactor designed according to the principle of aerodynamics is widely used in the separation of atmospheric particles with good results [16], and it is feasible to collect fungal spores using a microfluidic chip with a virtual impactor structure. This method has high collection efficiency and low cost [17,18]. In addition, microfluidic chip composite diffraction is used to detect and identify spores. For spores with large differences in size and shape, the identification accuracy is high. Compared with microscopic observation, its detection field of vision is much expanded, and the detection efficiency and speed are greatly improved [19,20]. However, the recognition accuracy of spores with similar shape and size is not high due to morphological detection. Therefore, it is necessary to design a separation and enrichment microfluidic chip with compound Raman spectroscopy to capture and collect spores and identify them accurately.

In this study, a two-stage separation and enrichment microfluidic chip with a semi arc pretreatment structure is proposed, which can be combined with micro Raman. Using numerical analysis and experiment, the best design parameters are obtained, and the feasibility of microfluidic chip is discussed. At the same time, micro Raman was used to detect the collected spores to obtain the Raman spectrum of fungal spores. The Raman spectrum was preprocessed and modeled for analysis. The fungal spores were identified, and their fingerprints were established to provide evidence for the subsequent prevention and treatment of fungal diseases.

## 2. Materials and Methods

### 2.1. Sample Preparation

In this study, *Ustilaginoidea virens* (*U. virens*), *Rice blast* (*R. blast*), *Aspergillus niger* (*A. niger*), and *Aspergillus carbonarius* (*A. carbonarius*) were taken as the research objects, among which the size of *R. blast* spores, *A. niger* spores, and *A. carbonarius* spores was 3–5 μm. The shape is similar to oval or round, and the spores of rice false smut are pear-shaped, with a size of 6–8 μm. The spores of *R. blast* and *U. virens* were provided by China Rice Research Institute in Hangzhou, China, and the spores of *A. niger* and *A. carbonarius* were purchased from Beijing Biological Preservation Center. Four fungi were cultured on PDA medium at 28 °C for 7 days, and mature spores with high activity and consistent activity were obtained. During the experiment, fresh conidia on PDA were scraped off and diluted with sterile distilled water in a sterile environment.

### 2.2. Composite Micro Raman Detection Method for Microfluidic Chips

**Figure 1:**
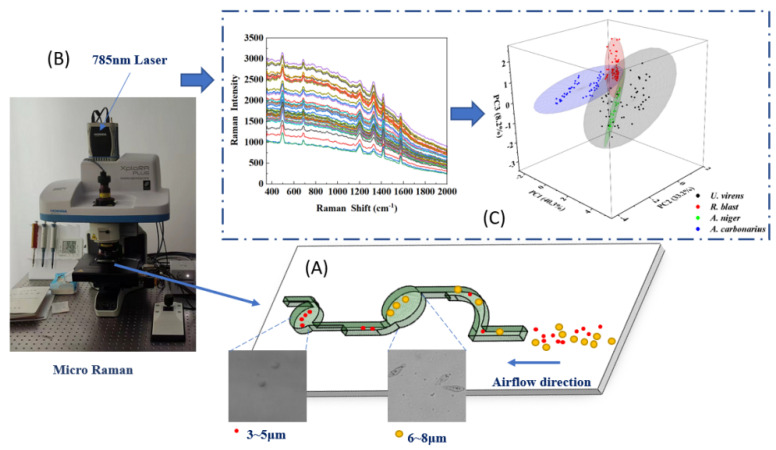
Detection of crop disease spores by microfluidic chip combined with micro Raman spectroscopy: (**A**) spore capture, separation, and enrichment; (**B**) micro Raman collection; (**C**) Raman data processing and modeling.

The microfluidic chip composite micro Raman detection method proposed in this study has the characteristics of rapidity, accuracy, and simple operation. As shown in Figure 1, this method mainly consists of three parts: spore capture, separation and enrichment, micro Raman collection, and Raman data processing and modeling. In this study, nanospheres (3–8 μm) were mixed with four spore suspensions; the four kinds of spores were *U. virens*, *R. blast*, *A. niger*, and *A. carbonarius*. The spore concentration in the real environment is very low, and there will be disease spores in the air unless the disease is about to occur. Therefore, in order to simulate the real experimental environment, the spores were mixed into an aerosol generator and an aerosol was generated, and the aerosol was released into the air. In order to simulate the real use environment, the spore concentration in the air was consistent with the concentration before the outbreak of a disease, 200 spore /$m^{3}$ [21]. An air pump was used to pump air into the microfluidic chip at the exit of the chip for about 3 min per acquisition. After the aerosol entered the microfluidic chip, the enrichment of 3–4 μm and 6–8 μm spores and impurities was completed through the two enrichment areas of the chip. The particles larger than 8 μm remained in the pretreatment channel due to their large inertia. The target particles entered the corresponding two enrichment areas, while the particles smaller than 3 μm flowed out of the chip through the outlet.

In this study, a total of 50 sets of Raman spectra of 4 species of spores were collected, and the collected 200 sets of Raman spectra were randomly divided into training set and test set at a ratio of 3:1. The samples were collected with a frequency shift of 200–2000 ${\mathrm{cm}}^{-1}$ using an XploRA PLUS Raman microscope (HORIBA, France), and the collected samples had 1053 features. The spectral acquisition parameters were set as follows: the excitation power was set to 365 mW, the 50 times objective lens was selected, spot size was 5 μm. Before the Raman spectrum was collected, the Raman spectrometer needed to be calibrated with wave number to eliminate the significant difference between the instrument response and the measured Raman spectrum value on the wave number axis and the true value. Wavenumber calibration requires measurement of the reference Raman spectrum from a standard material with well-defined Raman bands. In this study, the Raman spectrum peak of silicon wafer was selected to calibrate the spectrometer. When the first-order Raman spectrum peak of silicon wafer was located at the 520.7 ${\mathrm{cm}}^{-1}$ frequency shift, the instrument was calibrated. In the laser illumination channel, the narrowband single-mode continuous light with the wavelength of 785 nm was selected as the excitation light of Raman scattering. This is because the 785 nm excitation light can effectively reduce the background spontaneous fluorescence noise and improve the signal-to-noise ratio of the collected Raman signal.

In this study, spectral data were randomly divided into training set (140) and test set (60). Spore identification model was established according to the test set. Before data analysis, the collected Raman spectra were preprocessed by SG smoothing and SNV, and the iterative polynomial fitting method was used for baseline correction to eliminate the interference of baseline drift and spectrum noise of Raman spectra.

The principle of polynomial replacement fitting is to continuously compare and adjust the original spectral data during polynomial replacement fitting, and directly compare the adjusted spectral data with the points on the fitting curve. The advantage of baseline correction with this method is to gradually adjust the coefficients of the polynomial so as to gradually approach the actual baseline shape, and the calculated baseline function form is closer to the actual baseline [22]. Standard normalized variate (SNV) algorithm refers to a deviation method to standardize variable values. Through the numerical standardization and transformation of the original variables, the transformation results will eventually fall within the range of [0, 1]. The premise of SNV algorithm is that all wavelength variables present normal distribution, and then they are standardized. The main way to remove noise is to remove light scattering [23].

Principal component analysis (PCA) is an algorithm for dimension reduction of data features. Spectral data were transformed from high to low dimensions by linear variation. Low-order principal components were retained and high-dimensional and invalid information was removed, reducing the data dimension. Using the most relevant low-dimensional data for classification identification can effectively reduce the difficulty and complexity of data analysis [24,25]. Usually, PCA needs to retain the principal components to make the variance contribution rate reach more than 85%. In this study, all PCAs with cumulative contribution rate greater than 95% were selected. The stability compatible reweighted sampling (SCARS) algorithm measures the magnitude of the stability of a variable, and the larger the stability value, the more likely the variable is to be selected, and the more consistent the bands selected at each iteration. This enables guaranteeing stable and rapid variable selection. The principles of the SCARS algorithm are to take each wavelength as one individual, use the adaptive reweighted sampling and exponentially revealing function to remove regression coefficients, take band points with small weights from the partial least squares model, pick out band points with large stable values, and retain the subset with the lowest RMSECV for interaction validation to find the optimal combination of variables with high efficiency [26].

SVM is a supervised machine learning method based on finite sample statistical learning theory. According to the structural risk minimization (SRM) principle, small samples and nonlinearity can be solved by constructing an optimal classification hyperplane in high-dimensional space. BPANN is a powerful learning algorithm that enables highly nonlinear mapping between inputs and outputs by training sample data, constantly modifying network weights and thresholds to minimize the error function in the direction of a negative gradient, ultimately approaching the expected output [27].

### 2.3. Chip Design and Simulation

To realize the purification and enrichment of fungal disease spores, a two-stage separation and enrichment microfluidic chip with arcuate pretreatment channel was designed. When a particle enters the inertial impactor with accompanying air, its trajectory is related to the size of the particle. Some small mass particles can cross stream lines and be separated. However, other small mass particles can flow away with the deflection of airflow. This behavior of particles in the curved channel can be characterized by Stokes number [17,28].

(1) $$stk=\frac{\rho_{p}d_{p}^{2}C_{c}Q}{9\mu W}$$

where $d_{p}\;$ is the particle size (m), $\rho_{p}$ is the particle density (1000 kg $m^{-3}$), μ is the air viscosity ($1.81\times 10^{-5}\;N\cdot s\cdot m^{-2}$), Q is the air velocity at the inlet of the microfluidic device (m · $s^{-1}$), and W is the nozzle width (m). $C_{c}$ is the Cunningham sliding correction coefficient based on particle size, which can be obtained by Equation (2) [16]:

(2) $$C_{C}=1+\frac{2A\lambda }{d}+\frac{2Q\lambda }{d}e^{-\frac{bd}{2\lambda }}$$

where A = 1.234, Q = 0.413, b = 0.904, and λ is the average free path of an air molecule with a value of 6.95 ×$\;10^{-8}\;$ m. Thus, according to Equation (3) it can be reduced to

(3) $$C_{C}\approx \{\begin{array}{c} 1+2.52\frac{\lambda }{d},d>2\lambda \\ 1+3.29\frac{\lambda }{d},d<2\lambda \end{array}$$

Furthermore, ${stk}_{50}$ represents a Stokes number corresponding to a particle collection efficiency of 50%, which can be rearranged by Equation (1):

(4) $$d_{50}=\sqrt{\frac{9\mu W_{stk_{50}}}{\rho_{p}C_{c}V}}$$

The $d_{50}$ is defined as the cut-off size of the particles producing 50% collection efficiency at each impact stage, and for this study, *U. virens*, *R. blast*, *A. niger*, and *A. carbonarius* collected at $d_{50}$ were set as 3–5 μm and 4–6 μm to obtain the up-to-size W for the two separated enrichment stages of the chip.

**Figure 2:**
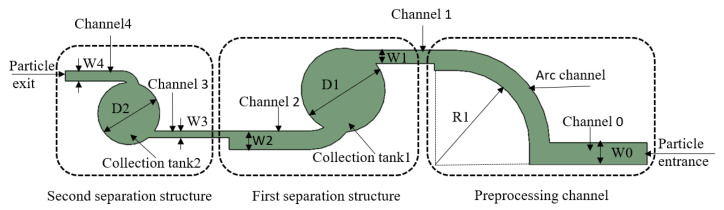
A 2D diagram of the microfluidic chip.

The microfluidic chip consists of an arc-shaped preprocessing channel and a two-stage separation and enrichment structure, and the particles enter the chip inlet jointly with air, then enter the preprocessing channel; the particles bonded together will be scattered and then enter the first enrichment area, and the remaining particles enter the second enrichment area due to the constant velocity at the inlet, while the particles that have a wide width channel enter the narrow width channel, so the airflow velocity is elevated, using velocity variation to manipulate particle enrichment versus rounding, which greatly increases collection efficiency. The structure of the microfluidic chip is schematically shown in Figure 2.

The preprocessing channel includes particle inlet, channel 1, and arc channel. The first separation structure includes channel 1, collection area 1, and channel 2. The second separation structure includes channel 3, enrichment zone 2, channel 4, and particle outlet. R1 is the radius of the pretreatment channel, and D1 and D2 are the diameters of the collection area, which are 5000 μm and 3700 μm, respectively. The length of channel 0 is 7000 μm, and the width is set to W0 = 1300 μm. Channel 1 has a length of 3500 μm and a width of 800 μm. Channel 2 has a length of 5650 μm and a width of 1100 μm. The length of channel 3 was set to 4400 μm, and the width was set to 400 μm. The length of channel 4 was set to 3600 μm, and the width was set to 600 μm.

### 2.4. Numerical Simulation

The numerical analysis software COMSOL multiphysics5.5 was used for simulation analysis in this study. The laminar flow module and particle trajectory tracking module in the software were used to simulate the separation process. The particle trajectory tracking module couples the effect of multiple force fields on the particle trajectory, calculating the trajectory of particles in the channel.

First, a 2D sketch of the microfluidic chip was drawn by AutoCAD2021 and then imported into COMSOL multiphysics5.5. The model needed to be meshed before the analysis, and the meshing was finer to achieve a better simulation. According to the actual situation of the subject, the Reynolds number did not exceed 1000, so the fluid was set as laminar flow. The Reynolds numbers in collection area 1 and 2 were 173 and 346, respectively. The particle density was 1.05 g ${\mathrm{cm}}^{-3}$ and the aerodynamic diameter was 0.5~8 µm. The inlet flow rate was 12.5 mL $\min^{-1}$, and the wall was set to be slip-proof. During the simulation, the collisions between the particles and the walls of the microfluidic device were inelastic, and the particles stuck to the walls to calculate the particle collection rate. Particle tracking uses Newton’s law of motion to solve differential equations. The research object of this topic is the movement of spores in the air. The spores were mainly driven by drag force in the microfluidic chip. The particle tracking module was used to simulate the particle trajectory. The drag force satisfies Stokes’ law, and the temperature and absolute pressure are standard states. Then, the simulation boundary was, the corresponding entrance and exit were set, and 100 particles were released at the entrance each time to ensure the reliability of particle collection efficiency. Since the simulation of this study needs to study the laminar flow and particle motion state in the microfluidic chip, transient and steady-state solvers were configured. The steady-state solver was used to study laminar flow, and the transient solver was used to study the particle. In the case of motion, the steady-state calculation amount is small, and one can choose to solve it directly. The solver can choose PARDISO to meet the requirements, and then choose the multi-threaded nested analysis pre-sorting algorithm, and the configuration of the steady-state solver is completed. The transient solver is more complicated to solve, and it needs to be solved iteratively. The GMRES solver was selected to solve the trajectory of the particle.

### 2.5. Chip Making

**Figure 3:**
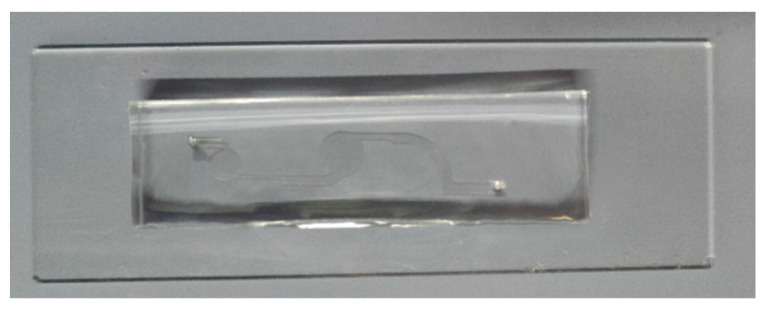
Chip physical map.

The microfluidic chip was fabricated using a conventional soft-lithography process. First, the photosensitive film was used to make a mask according to the structure drawn by AutoCAD2021, and the photosensitive film was covered on the copper plate to make the thickness reach 100 μm as the layer height of the channel. Then, ultraviolet lithography was used to expose the photosensitive film and a developing solution was used to develop it, so that only the punch of the microfluidic chip was left on the copper plate. Then, the outer mold was used to fix the channel chip range, and then the polydimethylsiloxane (PDMS) was poured into the mold. It was then put into a 70° oven to cure for 4 h, and finally the mold was taken out of the cured chip, and a plasma bonder was used to bond the chip and the glass slide to form a microfluidic chip. The physical map is shown in Figure 3.

## 3. Results and Discussion

### 3.1. Numerical Simulation of Microfluidic Chip

**Figure 4:**
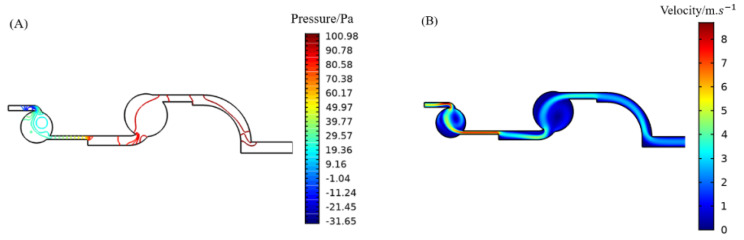
Microfluidic chip simulation diagram: (**A**) microfluidic chip pressure map; (**B**) microfluidic chip velocity map.

According to the set simulation conditions, it is necessary to simulate the parameters of the microfluidic chip to obtain a reasonable structure of the chip to obtain a good enrichment effect. First, the sub-bureau set the conditions to obtain the pressure map of the microfluidic chip in Figure 4A and the gas velocity map of Figure 4B in the microfluidic chip. In this study, the simulation of the channel width changed to obtain the best enrichment effect. The enrichment effect evaluation index is to release 100 particles, and the number of particles obtained in the corresponding enrichment area determines the enrichment efficiency. The number of particles can be counted in a specific area by selecting specific particles from the derived values in the simulation results of COMSOL multiphysics5.5, and the enrichment rate can be expressed as the percentage of the number of enriched particles to the total number of released particles.

**Figure 5:**
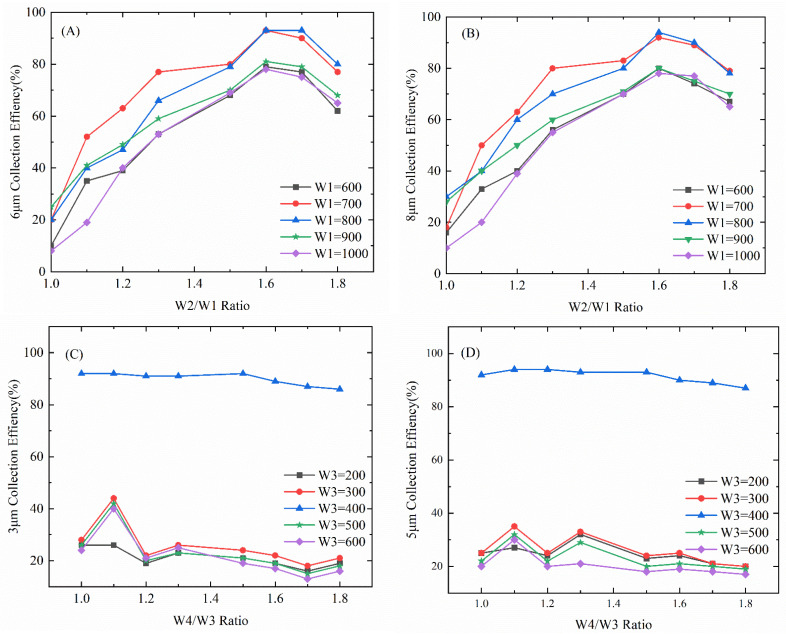
Statistical graph of particle enrichment imitation rate: (**A**) 6 μm collection efficiency; (**B**) 8 μm collection efficiency; (**C**) 3 μm collection efficiency; (**D**) 5 μm collection efficiency.

According to the simulation condition setting, and after simulation with the width adjustment of the microfluidic chip, the enrichment rate of the microfluidic chip under different ratios of the channel width was obtained. Figure 5A,B show the effect of the W2/W1 ratio on the separation and enrichment effect of particles, taking 6 μm and 8 μm particles as examples. It can be seen from Figure 4A,B that when W1 = 700 μm, the enrichment effect of 6 μm and 8 μm particles is better, and when W2/W1 = 1.6, the enrichment effect of the first enrichment zone is the best, the enrichment rate of 6 μm is 93%, and the enrichment rate of 8 μm is 93%. The enrichment rate was 94%, and the channel widths were fixed at W1 = 700 μm and W2 = 1120 μm. It can be seen from Figure 5C,D that when W3 = 400 μm, the enrichment effect of 3 μm and 5 μm particles is better, while W4/W3 is 1.1, the enrichment efficiency of 3 μm particles is 93%, and the enrichment rate of 5 μm particles is 94%; at this time the fixed channel widths were W3 = 400 μm and W4 = 440 μm.

**Figure 6:**
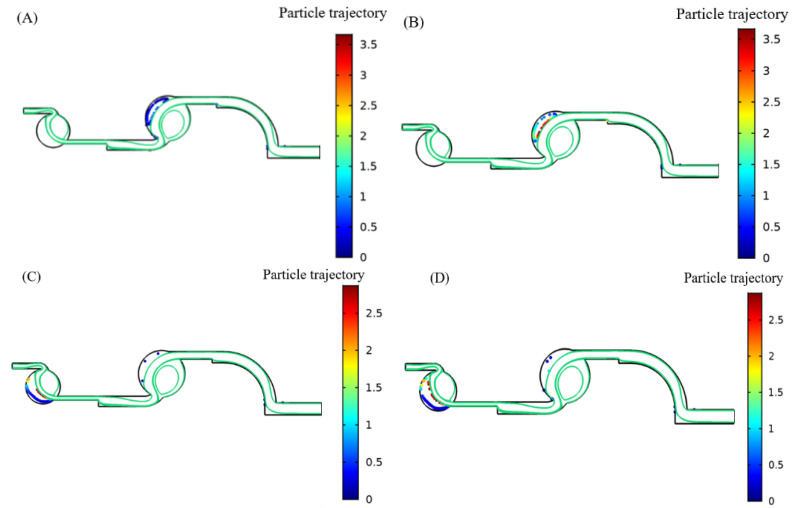
Statistical graph of particle enrichment imitation rate: (**A**) 6 μm collection effect; (**B**) 8 μm collection effect; (**C**) 3 μm collection effect; (**D**) 5 μm collection effect.

According to the above simulation and analysis, the optimal enrichment parameters of 6 μm, 8 μm, 3 μm, and 5 μm were obtained, and the best enrichment effect was obtained by simulation according to the optimal parameters, as shown in Figure 6.

### 3.2. Raman Analysis

**Figure 7:**
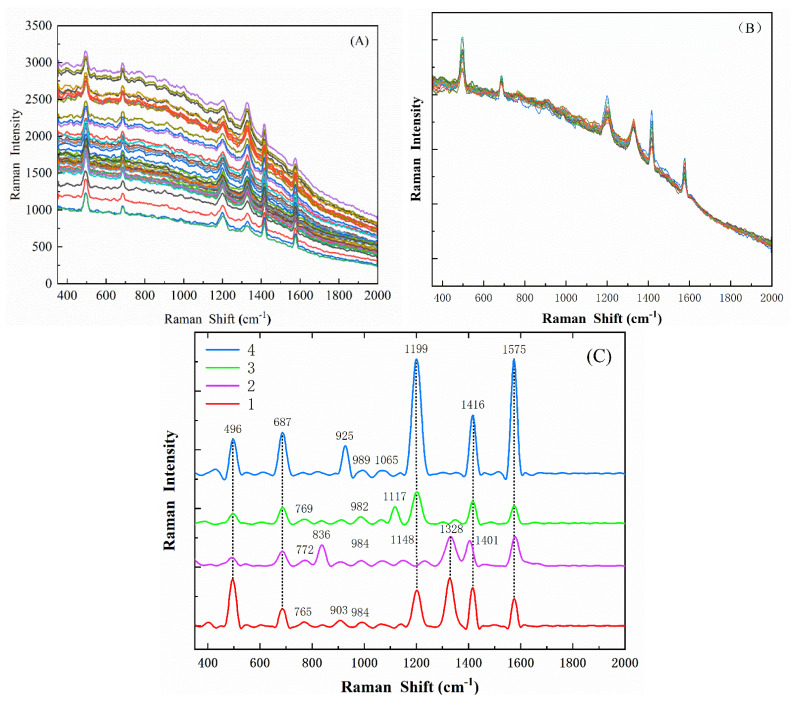
Raman analysis: (**A**) SG-smoothed spectrum after removing cosmic spikes; (**B**) spectra processed by SNV; (**C**) average Raman spectra of four diseased spores.

A total of 200 spectra of four fungi were collected in this study. Since the original spectral data have more spectral noise and high fluorescence background interference, effective spectral preprocessing is very important. Preprocessing the spectrum by selecting a reasonable spectral preprocessing method can effectively reduce spectral noise, retain useful information, simplify the modeling process, and improve the stability of the model. During the Raman spectrum acquisition process, the detector has a small probability of receiving various interference rays such as cosmic rays in the environment, forming sharp peaks in the spectrum, which affects the stability of the spectrum. Therefore, spectra with cosmic spikes need to be identified and eliminated before data analysis. This paper adopted SG smoothing and SNV to remove the influence of noise on the model. Among them, SG smoothing can eliminate the noise interference and uneven fluorescence intensity of the original Raman spectrum. As shown in Figure 7A, and SNV can construct an ideal spectrum by taking the average value of the spectrum, thereby eliminating the effect of particle scattering, as shown in Figure 7B. Baseline calibration is achieved by an iterative polynomial fitting method for baseline drift phenomena in the spectrum. The preprocessing steps are: (1) removing the cosmic spike Raman curve; (2) SG smoothing; (3) SNV correction; (4) baseline calibration.

It can be seen that the SNV greatly reduces the influence of baseline drift and noise on the spectra, while preserving the important spectral information of the fungus. Figure 7C shows the processed average spectra collected from four fungi to provide Raman fingerprints important for the identification of fungal cells. Since Raman scattering depends on the change in molecular polarizability during atomic vibrations, non-polar groups such as S–S, C–C, S–H, and N–N vibrations have strong corresponding signals in Raman, reflecting that various structural information of organic compounds has been obtained [29]. Diseased spores contain cell walls and abundant mRNA. The main components of cell walls are polysaccharides and a small number of proteins and lipids. Different spores contain different types of polysaccharides [30,31,32].

According to the existing research and experimental data, all characteristic spectral bands and spectral assignments of the four fungi are shown in Table 1. In all Raman spectra, the peaks at 493–497 ${\mathrm{cm}}^{-1}$ and 1416 ${\mathrm{cm}}^{-1}$ are characteristic peaks for galactomannan and chitin, which are important components of fungal cell walls [29,32]. The peak at 686–687 ${\mathrm{cm}}^{-1}$ is attributed to Guanine, Thymine [29,33]. The peaks at 765${\mathrm{cm}}^{-1}$–772 ${\mathrm{cm}}^{-1}$ were assigned to (O-P-O) stretching RNA, respectively [33]. The peak at 984${\;\mathrm{cm}}^{-1}$–989${\;\mathrm{cm}}^{-1}$ is attributed to C=C deformation, C–N stretching [12,29]. The peaks at 1065${\;\mathrm{cm}}^{-1}$ and 1117 ${\mathrm{cm}}^{-1}$ are galactomannan [29]. The peak at 1148 ${\mathrm{cm}}^{-1}$ was attributed to C–O ring aromatic [12]. The peaks at 1200 ${\mathrm{cm}}^{-1}$ and 1202 ${\mathrm{cm}}^{-1}$ are Amide III (random) and Thymine [32,33]. The peak at 1328 ${\mathrm{cm}}^{-1}$ is attributed to C–O Amide III (protein), C–H deformation [12]. The peak at 1570 ${\mathrm{cm}}^{-1}$–1577 ${\mathrm{cm}}^{-1}$ is Adenine, Guanine (ring stretching) [12,32]. The Raman signals of diseased spores have common components, and there are also differences with their own characteristics. Therefore, the Raman fingerprints of the four fungi measured in this study provide a basis for species identification.

Combining Figure 7 and Table 2, it can be concluded that the Raman spectra of the four disease spores have some significant common characteristic peaks, which indicates that they contain many of the same components, and there are also some distinctive characteristic peaks unique to spores, indicating their unique composition. However, there were also some insignificant shared characteristic peaks and unique characteristic peaks, which also indicated some shared and unique compositions of diseased spores. Then, classification modeling of spores by only significant characteristic peaks led to inaccurate classification of spores, and it was necessary to find all characteristic peaks by training the algorithm on all bands of the Raman spectrum.

### 3.3. Fungal Spore Recognition Model

The large number of Raman spectral features and the existence of a large number of redundant features greatly slow down the speed of modeling and analysis, so dimensionality reduction was required before modeling and analysis. After dimensionality reduction, the Raman spectrum was modeled and analyzed by SVM and BPANN, and a classification model was established.

**Figure 8:**
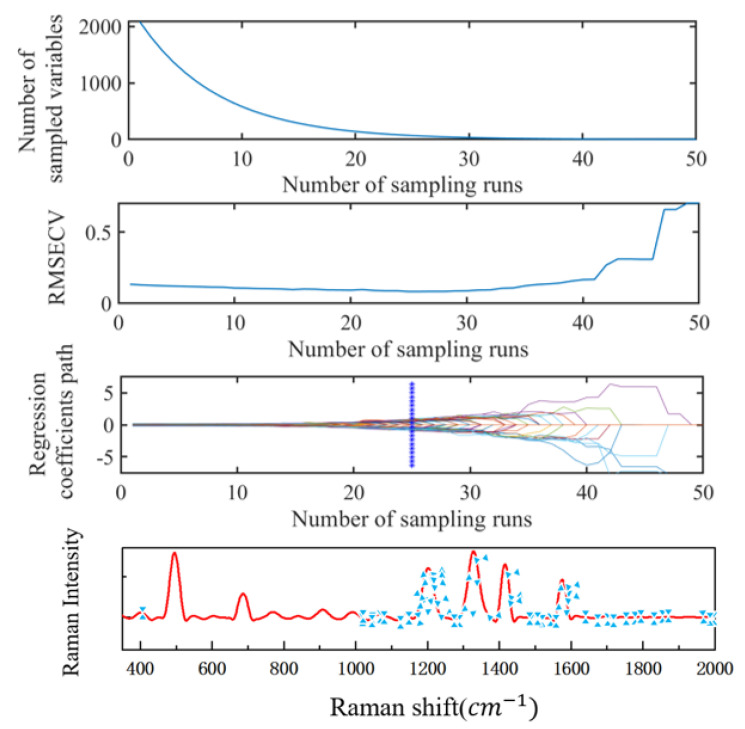
Example of running result of SCARS algorithm.

The SCARS algorithm uses the stability of the variable as a measure. The greater the stability value, the greater the possibility of the variable being selected, and the frequency bands selected for each iteration can be consistent. It can ensure the stability and speed of variable selection. The optimal potential frequency band variable is selected by the Monte Carlo cross-validation method, and an RMSECV value can be obtained in each cycle. Due to the large number of sampling times, in order to obtain a better combination of characteristic frequency bands, it is necessary to compare through repeated trials. The subset combination corresponding to the minimum RMSECV is obtained. When the number of cyclic samplings is set to 25, the running result tends to be stable. The running result of the algorithm is shown in Figure 8. The algorithm filtered out 69 bands, as shown in Figure 8. 

**Figure 9:**
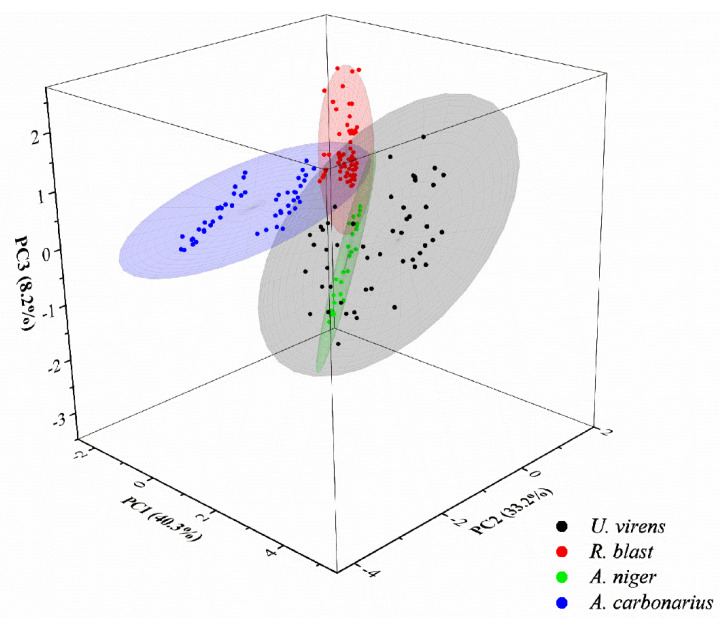
Top 3 PCA distribution results.

PCA selects different PCA variables in full and shortened spectral intervals. The top 15 PCAs are the most significant for the raw spectral data, with a cumulative contribution rate of over 95%. Figure 9 shows the top three PCA taxonomic groups of fungi and observed preliminary taxonomic results for four diseased spores.

After the disease spore Raman spectrum was dimensionally reduced, the data were input into the classification model for classification. The data after dimensionality reduction by PCA is a matrix of 200 × 15, and the data after dimensionality reduction by SCARS is a matrix of 200 × 65. In this study, two excellent classification algorithms were selected to classify the dimensionality-reduced data. When the SVM was running, the radial basis function (RBF) kernel function was selected, and then the optimal parameters of the penalty function c of the model and the kernel parameter g of the kernel function were obtained through grid search. The optimal parameters are 0.00094, and the accuracy rates of the test set and prediction set are 94.38% and 86.63%. When using BPANN, the hidden layer transfer function was set to Tansig, the output layer transfer function to purelin, the network training function to trainbfg, the number of training iterations to be 1000, and the target error to be 0.0001 [29]. The discriminative accuracies of the training set and prediction set are 88.46% and 87.61%, respectively. Then, the Raman data reduced by PCA and SCARS were used to classify the classification model. Since the random division of the test set and the prediction set and other factors will affect the classification effect of the pull model, the model was run five times and its average accuracy was used. The results are shown in Table 2. The calibration set and prediction set of SCARS-BPANN classification have the highest accuracy, 94.94% and 94.31%, respectively.

### 3.4. Enrichment Experiment

**Figure 10:**
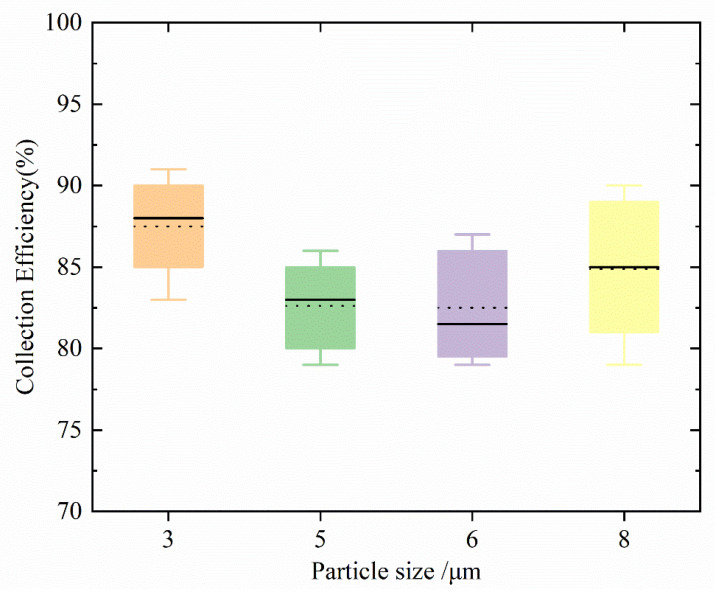
Enrichment Experiment Statistics Box Plot.

In order to verify the enrichment effect of the microfluidic chip determined by the simulation, a quantitative number of nano-microspheres was sucked into the chip inlet, and then the enrichment efficiency of the particles in the chip was observed through a microscope. About 50 nano-microspheres of one particle size were mixed into the aerosol generator each time, all of them were entered from the chip inlet and out, and then the enriched particles were counted by a microscope and the enrichment rate was calculated. The particle size was tested five times, and the statistical boxplot of the enrichment experiment as shown in Figure 10 was obtained. The average enrichment efficiencies of 3 μm, 5 μm, 6 μm, and 8 μm were 87.5%, 82.625%, 82.5%, and 84.87%, respectively. It can be seen from Figure 10 that the enrichment rate obtained in the enrichment experiment is slightly lower than that obtained by simulation, but the enrichment efficiency is stable.

### 3.5. Discussion

Airborne fungal diseases mostly float in the air in the form of spores and spread with the wind before their widespread outbreaks [4,8]. At the time of disease occurrence, the concentration of disease spores in the air is higher than 100 spores /$m^{3}$; if timely detection and prevention and control measures are not taken, the disease spores will drift to other areas with the wind and continue to infect other areas. Y. Zhang et al. proposed a deep-learning-based fungal spore detector FSNet for recognition and automatic counting of *Aspergillus glaucus, Penicillium solitum*, and *Aspergillus candidus*, and the experiments demonstrated that FSNet achieved an average precision of 0.9, 0.944, and 0.904 on *Aspergillus glaucus, Penicillium solitum,* and *Aspergillus candidus*, respectively, demonstrating the ability to automate detection of spores in the laboratory [7]. However, the automatic detection of spores based on images cannot accurately identify spores with similar appearance. Aswathi S. et al. were able to differentiate between dead and live *C. sporogenes* spores on media (SBA and TSA) plates using hyperspectral imaging [34]. The use of hyperspectral images and spectral information can accurately identify spores, but the effective reflectance of the hyperspectral spectrum for spores is limited to the spectral band range, and the spores cannot be captured and detected. The method for detecting and identifying disease spores of microfluidic chip combined with Raman microscopy developed in this study can capture spores in the air and then accurately identify the spores by identifying their Raman fingerprints, and this method does not require cumbersome biochemical experiments with low cost.

## 4. Conclusions

In this study, a two-stage separation and enrichment microfluidic chip with an arc-shaped pretreatment channel was developed and designed to separate and enrich crop disease spores, and then combined with confocal Raman microscopy to conduct Raman fingerprinting of disease conidia. The map was accurately identified. Support vector machine (SVM) and back-propagation artificial neural network (BPANN) were used to identify fungal spore species, and the identification accuracy was 86.32% and 87.61%; the SCARS-SVM model had the best discriminant effect, with a discriminant accuracy of 94.31%. Therefore, the capture and identification method of crop disease spores based on microfluidic chip and micro Raman may become an accurate, convenient, and inexpensive method for detection and identification of fungal spores.

## Figures and Tables

**Table 1:** Peak assignment of the average spectrogram of the four spores.

Raman Shift ($cm^{-1}$)	*U. virens*	*R. blast*	*A. niger*	*A. carbonarius*	Tentative Assignments	Reference
493–497	496	493	497	497	Galactomannan, chitin	[29]
686–687	686	686	687	687	Guanine, Thymine (ring breathing)	[29,33]
765–798	765	772	769	769	(O-P-O) stretching RNA	[33]
930–990	984	984	982	989	C=C deformation, C–N stretching	[12,29]
1065–1117	-	-	1117	1065	galactomannan	[29]
1150–1185	1148	1148	-	-	C–O ring aromatic amino acid in protein	[12]
1200–1274	1202	-	1202	1200	Amide III (random), Thymine	[32,33]
1315–1325	1328	1328	-	-	Amide III (protein), C–H deformation	[12]
1416	1416	1401	1416	1416	Chitin	[32]
1570–1595	1575	1577	1575	1575	Adenine, Guanine (ring stretching)	[12,32]

**Table 2:** Accuracy statistics of spore classification model.

Serial Number	Algorithm	Calibration Set Accuracy (%)	Prediction Set Accuracy (%)
1	SVM	85.65	86.32
2	BPANN	88.46	87.61
3	PCA-SVM	90.25	91.24
4	PCA-BPANN	88.34	87.55
5	SCARS-SVM	93.41	93.43
6	SCARS-BPANN	94.94	94.31

## Data Availability

Data is contained within the article.
